# Cardiac Troponin Elevation Beyond Type 1 Myocardial Infarction: A Systematic Review of Prognostic Significance and Cardiovascular Risk Stratification

**DOI:** 10.7759/cureus.112972

**Published:** 2026-07-19

**Authors:** Mohammad Abuzenah, Rabia Zameer, Sahil Kumar, Zaid Al Ghananeem, Palak Batra, Kaleem Khaan

**Affiliations:** 1 Internal Medicine, Northern Care Alliance NHS Foundation Trust, Salford, GBR; 2 Internal Medicine, Larkin Community Hospital South Miami, South Miami, USA; 3 Cardiology, National Institute of Cardiovascular Diseases, Karachi, PAK; 4 Internal Medicine, Aneurin Bevan University Health Board, Newport, GBR; 5 Internal Medicine, Ghulam Muhammad Mahar Medical College, Sukkur, PAK; 6 Internal Medicine, Mayo Hospital, Lahore, PAK

**Keywords:** cardiac troponin, heart failure, high-sensitivity troponin, myocardial injury, type 2 myocardial infarction

## Abstract

Cardiac troponin elevation is central to the diagnosis of myocardial injury, but its clinical interpretation remains challenging when type 1 myocardial infarction is absent. This systematic review evaluated the clinical and prognostic significance of elevated cardiac troponin in adults without type 1 myocardial infarction, with particular emphasis on type 2 myocardial infarction, critical illness-related myocardial injury, suspected acute coronary syndrome populations with minor myocardial injury, and acute or chronic heart failure-related myocardial injury. A systematic search of PubMed/MEDLINE, Scopus, and Web of Science identified six eligible clinical studies. The included evidence comprised prospective and retrospective cohort studies, secondary biomarker analyses of randomized trials, and trial-derived heart failure cohorts. Across heterogeneous clinical settings, troponin elevation was consistently associated with adverse outcomes, including all-cause mortality, cardiovascular mortality, heart failure hospitalization, cardiovascular death or heart failure rehospitalization, and greater clinical severity. In type 2 myocardial infarction and critical illness, troponin elevation identified high-risk patients despite the absence of classic plaque-rupture acute coronary syndrome. In heart failure populations, troponin appeared to function as a dynamic marker of myocardial stress, residual risk, and treatment-responsive injury. High-sensitivity assays further suggest that troponin may convey prognostic information across a continuum rather than only above binary diagnostic thresholds. These findings support a phenotype-based approach in which troponin elevation outside type 1 myocardial infarction is interpreted according to mechanism, clinical context, biomarker trajectory, and follow-up needs. Although current evidence does not define a uniform treatment pathway, it indicates that troponin-positive patients without type 1 myocardial infarction should not be considered low risk solely because acute coronary syndrome has been excluded. Future prospective studies should determine which troponin-positive phenotypes benefit most from structured cardiovascular reassessment, targeted investigation, and post-discharge surveillance.

## Introduction and background

Cardiac troponins I and T are regulatory proteins involved in cardiomyocyte contraction that enter the circulation when myocardial cells are injured. Conventional assays detect troponin at higher concentrations, whereas high-sensitivity assays reliably measure substantially lower concentrations and permit earlier recognition of myocardial injury. Cardiac troponin is therefore central to the contemporary evaluation of patients with suspected myocardial infarction, particularly with the widespread adoption of high-sensitivity assays. Myocardial injury is defined by a cardiac troponin concentration above the assay-specific 99th percentile upper reference limit and is considered acute when serial measurements demonstrate a rise or fall. Myocardial infarction requires acute myocardial injury accompanied by clinical evidence of myocardial ischemia; type 1 myocardial infarction specifically results from an acute atherothrombotic coronary event [1-3].

Troponin elevation is frequently encountered in emergency, inpatient, and critical care populations, although its reported prevalence varies considerably according to the clinical setting, assay used, and diagnostic threshold. A substantial proportion of troponin-positive patients do not have type 1 myocardial infarction, creating a large and clinically heterogeneous population for whom subsequent investigation, treatment, and follow-up remain uncertain [2-5]. Troponin elevation may occur in type 2 myocardial infarction, acute or chronic heart failure, sepsis, critical illness, renal dysfunction, tachyarrhythmia, pulmonary disease, and systemic oxygen supply-demand imbalance. This distinction is clinically important because myocardial injury and myocardial infarction are related but not interchangeable concepts [2,3].

The diagnostic framework for myocardial injury has improved substantially, yet uncertainty remains regarding the clinical response to troponin elevation once type 1 myocardial infarction has been excluded. In routine practice, a troponin-positive result often initiates an acute coronary syndrome (ACS) pathway. However, if plaque rupture or obstructive coronary thrombosis is not supported by the clinical presentation, electrocardiography, imaging, or serial biomarker pattern, the result may be treated as secondary, nonspecific, or incidental [4,5]. This approach may be understandable during acute triage, but it risks overlooking the prognostic information carried by troponin elevation. Even when the underlying mechanism is not acute coronary thrombosis, myocardial injury may reflect a vulnerable myocardium, advanced comorbidity burden, hemodynamic stress, occult cardiovascular disease, or residual post-discharge risk [6].

A clinically meaningful interpretation of troponin, therefore, requires more than a binary distinction between ACS and no ACS. Type 2 myocardial infarction, critical illness-related myocardial injury, minor myocardial injury in suspected ACS pathways, and heart failure-related troponin elevation represent different phenotypes with distinct mechanisms, baseline risks, and management implications [7,8]. At the same time, these entities share an important feature: troponin elevation may identify patients at increased risk of mortality, heart failure hospitalization, cardiovascular events, or treatment-relevant myocardial stress. The principal knowledge gap is whether and how this prognostic signal should inform cardiovascular risk stratification, clinical follow-up, and selective investigation without promoting unnecessary invasive testing or inappropriate ACS-directed therapy [6,7].

Accordingly, this systematic review addresses the following research question: among adults without type 1 myocardial infarction, what is the clinical and prognostic significance of cardiac troponin elevation across distinct myocardial injury phenotypes? Specifically, it examines evidence from type 2 myocardial infarction, non-ACS critical illness, suspected ACS populations with minor myocardial injury, and acute or chronic heart failure-related myocardial injury. The objective is to determine whether troponin elevation functions as a marker of cardiovascular vulnerability within each setting, how it relates to adverse outcomes, and how it may inform a more structured, phenotype-based approach to risk stratification, management, and follow-up.

This work has not been previously presented as a conference abstract, poster, oral presentation, meeting abstract, or scientific presentation.

## Review

Materials and methods

Study Design

The review protocol was not prospectively registered in PROSPERO or another public registry. This was due to an oversight during the initial planning stage, and retrospective registration was not pursued because data extraction and synthesis had already commenced. Nevertheless, the review was conducted using predefined eligibility criteria, outcomes, screening procedures, data-extraction methods, and risk-of-bias assessment tools in accordance with Preferred Reporting Items for Systematic Reviews and Meta-Analyses (PRISMA) 2020 [9]. The absence of prospective protocol registration is acknowledged as a limitation of the review.

Population, Invention, Comparison, and Outcome (PICO) Framework

The population of interest included adult patients with elevated cardiac troponin in the emergency department, inpatient, intensive care, or heart failure settings. The exposure was cardiac troponin elevation, high-sensitivity cardiac troponin elevation, type 2 myocardial infarction, acute or chronic myocardial injury, or troponin-defined myocardial stress in the absence of definite type 1 myocardial infarction. Eligible comparators included troponin-negative patients, patients with lower troponin concentrations, patients with type 1 myocardial infarction, different troponin strata, or treatment groups within randomized trial-derived biomarker analyses. Outcomes of interest included all-cause mortality, cardiovascular mortality, heart failure hospitalization, cardiovascular death or heart failure rehospitalization, adverse cardiovascular events, diagnostic discrimination, biomarker trajectory, treatment response, and risk prediction.

Search Strategy

A systematic literature search was conducted in PubMed/MEDLINE, Scopus, and Web of Science for studies published from January 1, 2015, through March 31, 2026. These complementary databases were selected to provide broad coverage of biomedical and multidisciplinary peer-reviewed literature. The search was intentionally concept-based and clinically broad because the review aimed to provide a qualitative synthesis across heterogeneous non-type 1 myocardial injury phenotypes rather than evaluate a single intervention, diagnosis, or narrowly defined population. Search concepts encompassed cardiac troponin biomarkers, myocardial injury and type 2 myocardial infarction, relevant non-ACS settings, prognosis, and clinical outcomes. Synonymous terms within each concept were combined using “OR,” and the principal concepts were linked using “AND.” Database-specific controlled vocabulary, field tags, phrase searching, truncation, and syntax were adapted to the indexing requirements of each platform. Where appropriate, exclusion terms were applied cautiously to reduce retrieval of studies restricted to type 1 ST-elevation myocardial infarction (STEMI) or perioperative myocardial injury without excluding potentially relevant mixed clinical populations. Table 1 therefore presents the representative terms and common conceptual logic applied across all three databases rather than identical syntax, which was not transferable between platforms.

**Table 1:** Database-wise representative search framework. ACS, acute coronary syndrome; hs-cTn, high-sensitivity cardiac troponin; hs-cTnI, high-sensitivity cardiac troponin I; hs-cTnT, high-sensitivity cardiac troponin T; MI, myocardial infarction; MINS, myocardial injury after non-cardiac surgery; STEMI, ST-elevation myocardial infarction.

Database	Search terms	Boolean operators / search logic
PubMed/MEDLINE	Troponin terms: “cardiac troponin,” troponin, “high-sensitivity troponin,” hs-cTn, hs-cTnT, hs-cTnI. Myocardial injury/type 2 MI terms: “myocardial injury,” “acute myocardial injury,” “chronic myocardial injury,” “type 2 myocardial infarction,” “type 2 MI,” “troponin elevation,” “elevated troponin.” Context/outcome terms: “non-ACS,” “non acute coronary syndrome,” prognosis, prognostic, mortality, outcomes, “heart failure hospitalization,” “cardiovascular death,” “risk stratification,” “risk assessment,” management, “follow-up.”	Related terms were combined with OR. Troponin, myocardial injury/type 2 MI, non-ACS context, and prognosis/outcome terms were combined with AND. Exclusion terms were applied with NOT where appropriate.
Scopus	Same representative terms as above, adapted to title, abstract, and keyword fields where appropriate.	Synonymous terms were combined with OR, major search domains with AND, and exclusion terms with AND NOT where appropriate.
Web of Science	Same representative terms as above, adapted to topic-based searching where appropriate.	Synonymous terms were combined with OR, search domains with AND, and exclusion terms with NOT where appropriate.

Study Selection

All records retrieved from the database searches were imported into a reference management workflow, and duplicates were removed before screening. Two reviewers independently screened titles and abstracts against the eligibility criteria. Full-text articles were then assessed independently by the same reviewers to determine final eligibility. Disagreements were resolved through discussion and consensus, and a third reviewer was consulted when required. The study selection process was documented according to PRISMA 2020 principles, including identification, screening, eligibility assessment, and final inclusion of primary studies.

Eligibility Criteria

Studies were eligible for inclusion if they enrolled adult human participants and evaluated cardiac troponin elevation, high-sensitivity troponin concentration, type 2 myocardial infarction, or myocardial injury in a clinical setting not primarily defined by type 1 myocardial infarction. Eligible clinical settings included suspected myocardial infarction or suspected ACS populations in which type 2 myocardial infarction, minor myocardial injury, or future heart failure risk was specifically evaluated; critically ill patients with troponin elevation in the absence of ACS or definite acute myocardial infarction (AMI); and patients with acute or chronic heart failure in whom troponin was assessed as a marker of myocardial injury, disease severity, prognosis, or treatment response. Randomized controlled trials, secondary biomarker analyses of randomized trials, prospective cohort studies, retrospective observational studies, and diagnostic or prognostic cohort studies were considered eligible if they reported clinically relevant outcomes.

Studies were excluded if they focused exclusively on type 1 myocardial infarction, STEMI, plaque-rupture ACS, perioperative myocardial injury, post-surgical troponin elevation, isolated assay validation, laboratory-only biomarker performance, pediatric populations, animal studies, case reports, editorials, conference abstracts without extractable data, or studies in which troponin was not linked to clinical outcomes, risk prediction, prognosis, or management implications. Studies were also excluded if the troponin elevation was evaluated only as a diagnostic rule-in or rule-out tool for ACS without meaningful analysis of non-type 1 myocardial injury, prognosis, heart failure risk, or follow-up relevance. These criteria allowed inclusion of studies such as type 2 myocardial infarction cohorts, ICU patients with troponin elevation but no AMI, suspected ACS cohorts evaluating later heart failure hospitalization, and heart failure trials assessing troponin as a marker of residual risk or treatment-responsive myocardial stress.

Data Extraction

Data were extracted using a standardized form developed for this review. Extracted variables included author name, publication year, study design, country or clinical setting where available, population characteristics, sample size, troponin assay or troponin-related exposure, comparator or grouping strategy, primary outcomes, follow-up duration, principal findings, and relevance to cardiovascular risk stratification. For trial-derived biomarker analyses, the parent trial design and treatment comparison were recorded, but the focus of extraction remained on troponin as a marker of myocardial injury, prognosis, or treatment response. Data extraction was performed by one reviewer and checked by a second reviewer for accuracy and consistency.

Quality Assessment

Risk of bias was assessed using tools appropriate to the design and purpose of each study. Prognostic biomarker studies were assessed using the Quality in Prognosis Studies tool [10], with attention to study participation, attrition, prognostic factor measurement, outcome measurement, confounding, and statistical analysis. Non-randomized observational studies with treatment exposure were assessed using Risk Of Bias In Non-randomized Studies of Interventions (ROBINS-I) [11], particularly for confounding, participant selection, exposure classification, missing data, outcome measurement, and selective reporting. Diagnostic prediction modelling evidence was considered using Prediction model Risk Of Bias ASsessment Tool (PROBAST) [12] principles where applicable. For randomized trial-derived biomarker analyses, the randomized treatment comparison was interpreted in the context of the parent trial design, but the biomarker-outcome association was assessed primarily as prognostic evidence because the review question focused on troponin-defined risk rather than randomized treatment efficacy alone.

Data Synthesis

A narrative synthesis was performed because of clinical and methodological heterogeneity across the included studies. The included studies differed in population, troponin assay, timing of measurement, comparator group, clinical setting, and outcome definition, making quantitative meta-analysis inappropriate. Findings were therefore synthesized according to clinically meaningful phenotypes: type 2 myocardial infarction, non-ACS critical illness-related myocardial injury, suspected ACS populations with minor myocardial injury, and heart failure-related myocardial injury. Particular attention was given to whether troponin elevation identified higher-risk patients after type 1 myocardial infarction was excluded, whether risk appeared graded across troponin concentrations, and whether serial or post-discharge troponin measurement provided additional prognostic information.

Results

Study Selection Process

**Figure 1:**
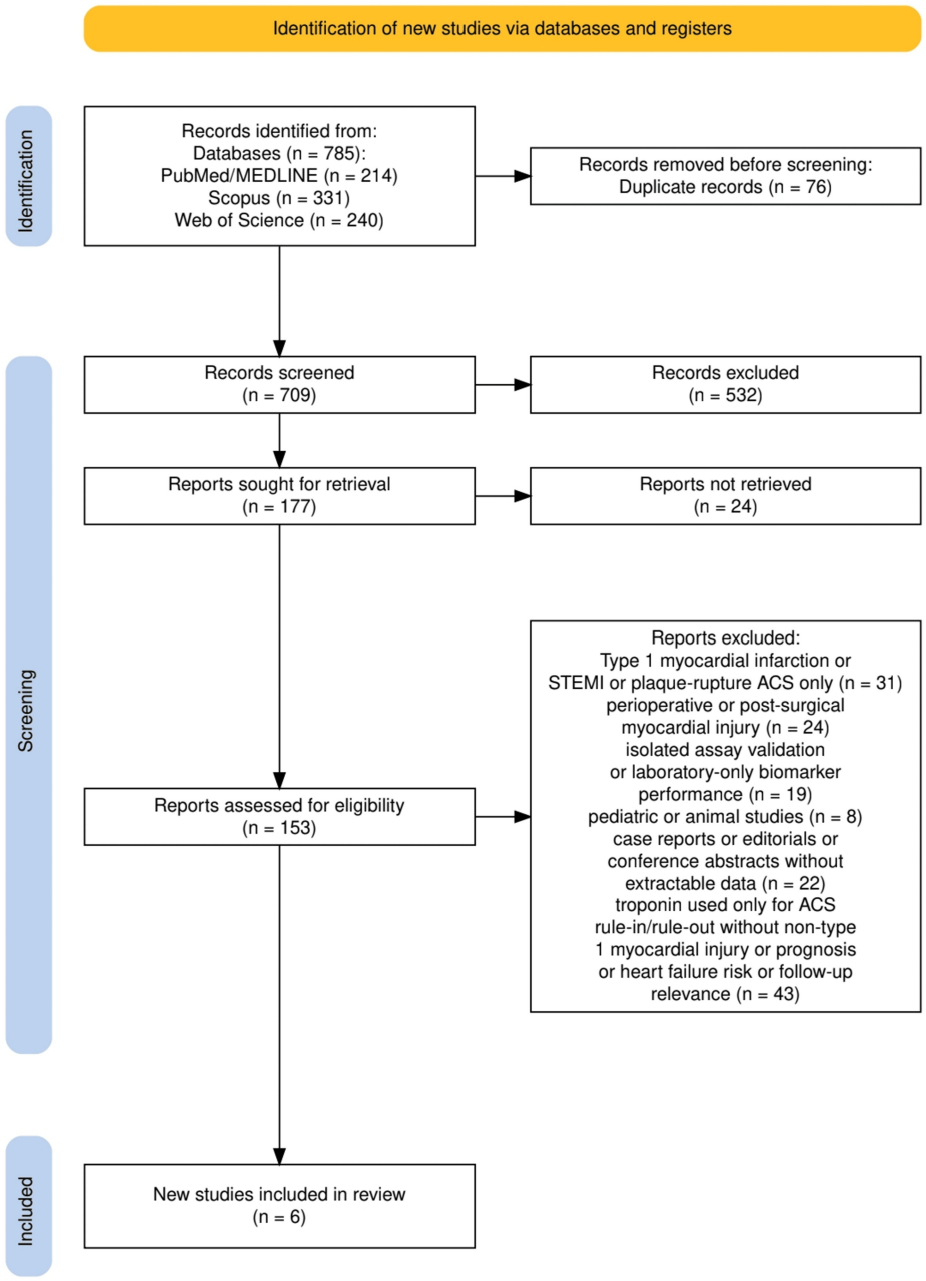
PRISMA flow diagram of study identification, screening, eligibility assessment, and inclusion. PRISMA, Preferred Reporting Items for Systematic Reviews and Meta-Analyses; STEMI, ST-segment elevation myocardial infarction; ACS, acute coronary syndrome

Figure 1 summarizes the PRISMA 2020 study selection process. The database search identified 785 records across PubMed/MEDLINE, Scopus, and Web of Science. After removal of 76 duplicates, 709 records were screened, of which 532 were excluded at the title and abstract stage. Of the 177 reports sought for retrieval, 24 could not be retrieved, leaving 153 full-text reports assessed for eligibility. A total of 147 reports were excluded for predefined reasons, most commonly because they focused only on type 1 myocardial infarction or STEMI, perioperative myocardial injury, isolated assay validation, non-extractable publication types, or ACS rule-in/rule-out without relevant prognostic or non-type 1 myocardial injury analysis. Ultimately, six studies were included in the final systematic review, as shown in Figure 1.

Characteristics of the Selected Studies

The characteristics of the included studies are summarized in Table 2. Overall, the evidence base comprised six clinical studies, including prospective and retrospective cohort analyses, secondary biomarker analyses of randomized controlled trials, and trial-derived heart failure cohorts. The included populations covered clinically relevant non-type 1 myocardial injury settings, including suspected myocardial infarction, critical illness without AMI, suspected ACS with minor myocardial injury, and acute or chronic heart failure with reduced ejection fraction (HFrEF). Sample sizes ranged from 597 to 4748 participants, and troponin exposure was assessed using conventional or high-sensitivity cardiac troponin assays. Across studies, outcomes included all-cause mortality, cardiovascular mortality, heart failure hospitalization, cardiovascular death or heart failure rehospitalization, diagnostic discrimination, biomarker trajectory, and treatment-related changes in myocardial stress.

**Table 2:** Characteristics and clinically relevant findings of included studies evaluating cardiac troponin elevation outside type 1 myocardial infarction. ACS, acute coronary syndrome; AMI, acute myocardial infarction; ASTRONAUT, Aliskiren Trial on Acute Heart Failure Outcomes; AUC, area under the curve; cGMP, cyclic guanosine monophosphate; EF, ejection fraction; EMPEROR-Reduced, Empagliflozin Outcome Trial in Patients With Chronic Heart Failure and a Reduced Ejection Fraction; HF, heart failure; HR, hazard ratio; hs-cTnI, high-sensitivity cardiac troponin I; hs-cTnT, high-sensitivity cardiac troponin T; ICU, intensive care unit; MI, myocardial infarction; ng/L, nanograms per liter; ng/mL, nanograms per milliliter; NYHA, New York Heart Association; PIONEER-HF, Comparison of Sacubitril/Valsartan Versus Enalapril on Effect on NT-proBNP in Patients Stabilized From an Acute Heart Failure Episode; sST2, soluble suppression of tumorigenicity 2; URL, upper reference limit.

Study	Population / setting	Troponin-related exposure	Comparator / grouping	Main outcomes assessed	Key clinically relevant finding
Neumann et al., 2017 [13]	Prospective cohort of 1548 patients with suspected myocardial infarction presenting in the emergency department; 99 patients had final diagnosis of type 2 MI	Type 2 myocardial infarction identified using clinical and high-sensitivity troponin I parameters	Type 1 MI vs type 2 MI; diagnostic score using female sex, absence of radiating chest pain, and baseline hs-cTnI ≤40.8 ng/L	Diagnostic discrimination between type 1 and type 2 MI; mortality follow-up up to 2 years; 1-year mortality	Type 2 MI patients had high 1-year mortality 13.8%, comparable to type 1 MI 9.4%. A simple clinical-laboratory score showed moderate discrimination for type 2 MI, with AUC 0.71.
Tsai et al., 2020 [14]	Retrospective observational study of 597 critically ill ICU patients hospitalized for >3 days in a tertiary teaching hospital; patients with definite AMI were excluded	Troponin I elevation on ICU admission, defined as >0.1 ng/mL	Elevated vs non-elevated troponin I; among troponin-positive patients: no antithrombotic prescription vs chronic prescription vs new prescription during ICU admission	30-day and 1-year all-cause mortality; association of antithrombotic use with outcomes	Troponin I elevation without AMI was common, present in 407/597 patients (68%), and was associated with higher 30-day mortality HR 1.679 and 1-year mortality HR 1.568. New antithrombotic prescription did not significantly improve outcomes, while chronic use was associated with better 1-year survival.
Stelzle et al., 2018 [15]	Cohort study of 4748 consecutive patients with suspected acute coronary syndrome presenting to three secondary/tertiary hospitals; mean age 61 ± 16 years, 57% male	High-sensitivity cardiac troponin I concentration, including values above and below the upper reference limit	Troponin above vs below upper reference limit; also assessed troponin concentration as a continuous predictor among patients below the URL	Subsequent heart failure hospitalization; predictive performance of troponin for heart failure risk	Patients with troponin above the URL had markedly higher heart failure hospitalization rates than those below the URL, 118/1000 vs 17/1000 person-years, with adjusted HR 7.0. Even below the URL, each doubling of troponin was associated with higher HF hospitalization risk, and adding troponin improved prediction, C-statistic 0.80 to 0.86.
Greene et al., 2018 [16]	Secondary analysis of the ASTRONAUT trial including 1469 patients hospitalized for heart failure with reduced ejection fraction, EF ≤40%, with pre-discharge troponin data	Troponin I elevation measured before discharge and at 1-month follow-up; elevation defined as >0.04 ng/mL	Pre-discharge elevated vs non-elevated troponin; 1-month elevated vs non-elevated troponin; persistent, newly elevated, or normalized troponin patterns	12-month all-cause mortality; cardiovascular mortality or heart failure hospitalization	Troponin elevation was common: 41.5% pre-discharge and 29.9% at 1 month. Pre-discharge elevation was not independently associated with 12-month outcomes, but 1-month troponin elevation predicted all-cause mortality HR 1.59 and cardiovascular mortality or HF hospitalization HR 1.28.
Packer et al., 2021 [17]	Secondary analysis of the EMPEROR-Reduced trial including 3636 patients with NYHA class II–IV heart failure with reduced ejection fraction randomized to empagliflozin or placebo	Baseline high-sensitivity cardiac troponin T measured in >97% of trial participants	Patients divided into four groups according to degree of hs-cTnT elevation; empagliflozin vs placebo across troponin strata	Cardiovascular death or heart failure hospitalization; renal outcomes; clinical severity; health status	Higher hs-cTnT levels were associated with greater clinical severity and worse prognosis. In placebo-treated patients, the highest hs-cTnT group had 3–5-fold higher risk of cardiovascular death and HF hospitalization than those with normal values. Empagliflozin improved HF and renal outcomes regardless of baseline hs-cTnT level.
Morrow et al., 2019 [18]	Biomarker analysis from the PIONEER-HF randomized double-blind trial including hospitalized patients with acute decompensated heart failure with reduced ejection fraction after haemodynamic stabilization; 694 patients had all baseline biomarkers	Circulating high-sensitivity troponin T as a marker of myocardial injury/stress, measured serially from baseline to 8 weeks	Sacubitril/valsartan vs enalapril; serial biomarker changes over time	Change in hsTnT, sST2, and urinary cGMP; exploratory association of biomarkers with cardiovascular death or HF rehospitalization	Sacubitril/valsartan produced greater reductions in hsTnT and sST2 than enalapril, with a 16% greater reduction in hsTnT at 4 weeks. Week-1 hsTnT and sST2 concentrations were associated with subsequent cardiovascular death or HF rehospitalization.

Quality Assessment

The risk of bias assessment is summarized in Table 3. Overall, the included studies demonstrated variable but generally acceptable methodological quality for a clinically oriented systematic review. The prospective and large trial-derived biomarker analyses had stronger internal validity because of structured participant selection, standardized biomarker measurement, and objective clinical outcomes, although several were secondary or exploratory analyses rather than studies primarily designed to test troponin-guided management. The retrospective ICU cohort was judged to have a moderate risk of bias because of potential residual confounding, treatment-selection bias, and single-centre design. Across studies, the main limitations included heterogeneity in clinical populations, differences in troponin assays and thresholds, variable timing of biomarker assessment, and incomplete ability to establish causality between troponin elevation and outcomes. Despite these limitations, the evidence consistently supported troponin elevation as a clinically relevant prognostic marker outside type 1 myocardial infarction.

**Table 3:** Risk of bias assessment of included studies evaluating cardiac troponin elevation outside type 1 myocardial infarction. ACS, acute coronary syndrome; AMI, acute myocardial infarction; ASTRONAUT, Aliskiren Trial on Acute Heart Failure Outcomes; CAD, coronary artery disease; EMPEROR-Reduced, Empagliflozin Outcome Trial in Patients With Chronic Heart Failure and a Reduced Ejection Fraction; HF, heart failure; HFrEF, heart failure with reduced ejection fraction; hs-cTnI, high-sensitivity cardiac troponin I; hs-cTnT, high-sensitivity cardiac troponin T; ICU, intensive care unit; MI, myocardial infarction; PIONEER-HF, Comparison of Sacubitril/Valsartan Versus Enalapril on Effect on NT-proBNP in Patients Stabilized From an Acute Heart Failure Episode; PROBAST, Prediction Model Risk of Bias Assessment Tool [12]; QUIPS, Quality in Prognosis Studies [10]; RoB 2, revised Cochrane risk-of-bias tool for randomized trials [19]; ROBINS-I, Risk Of Bias In Non-randomized Studies of Interventions [11].

Study	Study design / evidence type	Suitable risk of bias tool	Key risk of bias considerations	Overall judgment
Neumann et al., 2017 [13]	Prospective cohort / diagnostic prediction model for type 2 MI discrimination with prognostic follow-up	PROBAST for prediction model; QUIPS for prognostic component	Prospective enrolment and adjudicated MI classification strengthen validity. However, the diagnostic score had only moderate discrimination and requires external validation. Small number of type 2 MI cases, n=99, limits precision.	Moderate risk / some concerns
Tsai et al., 2020 [14]	Retrospective observational ICU cohort assessing troponin elevation and antithrombotic exposure	ROBINS-I	Clear non-ACS/AMI population and objective mortality outcomes are strengths. Limitations include retrospective design, single-centre setting, potential residual confounding by illness severity, and treatment-selection bias for antithrombotic prescription.	Moderate risk
Stelzle et al., 2018 [15]	Cohort study of suspected ACS patients assessing hs-cTnI and later HF hospitalization	QUIPS	Large consecutive cohort and objective HF hospitalization outcome support reliability. Residual confounding may remain because troponin elevation reflects underlying cardiovascular disease burden.	Low to moderate risk
Greene et al., 2018 [16]	Secondary biomarker analysis of ASTRONAUT trial in hospitalized HFrEF	QUIPS	Trial-derived cohort and core-lab troponin measurement are strengths. Main limitations are secondary analysis design, possible missing biomarker data, and confounding by HF severity or CAD status.	Moderate risk
Packer et al., 2021 [17]	Secondary biomarker analysis of EMPEROR-Reduced trial in chronic HFrEF	QUIPS for prognostic biomarker analysis; RoB 2 only for randomized treatment effect	Large randomized-trial population with hs-cTnT measured in >97% of participants. Prognostic association is graded and clinically coherent, but the biomarker analysis remains secondary and may be influenced by disease severity, renal function, and comorbidity.	Low to moderate risk
Morrow et al., 2019 [18]	Biomarker analysis from PIONEER-HF randomized trial in acute decompensated HFrEF	QUIPS for biomarker-outcome association; RoB 2 for treatment comparison	Randomized, double-blind trial setting and serial biomarker measurement strengthen internal validity. However, the hsTnT outcome association was exploratory and not the primary trial endpoint.	Moderate risk

Discussion

Principal Synthesis

This review found that cardiac troponin elevation outside type 1 myocardial infarction was consistently associated with adverse outcomes across several distinct clinical settings. These included mortality in type 2 myocardial infarction and critical illness, subsequent heart failure hospitalization in suspected ACS populations, and greater disease severity and cardiovascular risk in acute or chronic heart failure. However, the mechanisms, troponin kinetics, baseline risks, and clinical implications differed substantially among these phenotypes. Troponin elevation should therefore be interpreted as a context-dependent prognostic marker rather than as evidence of a single disease process. Although the findings support greater recognition of the risk associated with non-type 1 myocardial injury, they do not establish that troponin-guided investigation, treatment, or follow-up improves clinical outcomes.

Type 2 Myocardial Infarction as a High-Risk Phenotype

Type 2 myocardial infarction represents a clinically important high-risk phenotype. In the study by Neumann et al. [13], patients with type 2 myocardial infarction were difficult to distinguish from those with type 1 myocardial infarction and had substantial one-year mortality. Type 2 myocardial infarction is a mechanistic diagnosis caused by an imbalance between myocardial oxygen supply and demand rather than acute atherothrombosis [20]. Potential triggers include anemia, sepsis, tachyarrhythmia, hypoxia, hypotension, and severe hypertension. Its prognostic significance may reflect both the severity of the precipitating illness and underlying cardiovascular vulnerability. These findings support careful identification of the trigger and individualized assessment of cardiovascular risk; however, the available evidence does not demonstrate that a standardized cardiovascular investigation or follow-up pathway improves outcomes. Such strategies require prospective evaluation.

Mechanism and Prognosis

In critical illness, troponin elevation may result from hypoxia, sepsis, renal dysfunction, vasopressor exposure, tachyarrhythmia, microvascular dysfunction, or systemic oxygen supply-demand imbalance. These mechanisms should be distinguished from plaque rupture, but identifying a non-atherothrombotic cause does not eliminate the prognostic information conveyed by the biomarker. Tsai et al. [14] found that troponin I elevation among intensive care patients without AMI was associated with increased 30-day and one-year mortality. Nevertheless, this association may partly reflect greater illness severity and residual confounding rather than an independently modifiable cardiac process. Troponin elevation in this setting should therefore be interpreted alongside the underlying illness, clinical trajectory, electrocardiographic findings, and other evidence of cardiac dysfunction. Current evidence does not support routine ACS-directed therapy or a uniform cardiovascular management pathway for all critically ill troponin-positive patients.

Heart Failure Signal

In heart failure, troponin elevation appears to reflect ongoing myocardial stress and injury rather than infarction alone. Greene et al. [16] found that troponin elevation one month after hospitalization, but not necessarily before discharge, was associated with subsequent mortality and heart failure events, suggesting that persistent injury after clinical stabilization may identify residual risk. Packer et al. [17] demonstrated a graded association between high-sensitivity troponin T and adverse outcomes in chronic HFrEF. Morrow et al. [18] found greater reductions in high-sensitivity troponin T with sacubitril/valsartan than with enalapril following acute decompensated heart failure. Collectively, these findings suggest that troponin may reflect disease severity, persistent myocardial stress, and biomarker response to treatment. However, biomarker reduction cannot be assumed to mediate improved clinical outcomes, and the evidence does not establish troponin-guided treatment as an effective management strategy.

Continuous Risk

High-sensitivity assays challenge a purely binary interpretation of troponin. Although the 99th percentile upper reference limit remains central to defining myocardial injury, prognostic risk may extend across concentrations below this diagnostic threshold. Stelzle et al. [15] found that high-sensitivity cardiac troponin I was associated with subsequent heart failure hospitalization both above and below the upper reference limit. This suggests that the threshold used to define myocardial injury may not correspond precisely to the concentration at which future cardiovascular risk begins to increase. Nevertheless, a detectable or subthreshold troponin concentration is not, by itself, an indication for intervention. Its significance should be interpreted in relation to the clinical presentation and baseline cardiovascular risk, and the utility of incorporating subthreshold concentrations into management pathways requires prospective validation.

Phenotype-Based Framework

The heterogeneity of the included populations argues against treating all non-type 1 troponin elevations as a single clinical entity. A phenotype-based framework may instead distinguish type 2 myocardial infarction, acute or chronic myocardial injury, critical illness-related injury, and heart failure-related injury [5,7]. Type 2 myocardial infarction is interpreted in relation to the precipitating oxygen supply-demand imbalance and underlying cardiovascular disease [13,20]. Critical illness-related injury is considered alongside illness severity and recovery trajectory [8,14], whereas heart failure-related injury may reflect persistent myocardial stress and greater residual risk [15-18].

This framework is intended to organize clinical interpretation by linking troponin elevation to its probable mechanism and phenotype-specific prognosis. It should not be regarded as a validated management algorithm or as evidence that every troponin-positive patient requires additional testing or specialist follow-up. Whether phenotype-guided assessment improves clinical outcomes remains a hypothesis that should be tested prospectively.

Clinical Implications

The clinical implication of this synthesis is that troponin elevation outside type 1 myocardial infarction should not be managed as either ACS or a harmless laboratory abnormality. A more balanced approach is needed. Once plaque rupture has been considered and reasonably excluded, clinicians should ask what cardiovascular or systemic vulnerability the troponin signal has revealed [5,7]. This may include evaluation for heart failure, occult coronary disease, arrhythmia, renal dysfunction, uncontrolled hypertension, anemia, pulmonary disease, or systemic illness [6,8]. Importantly, risk recognition should not translate into indiscriminate antithrombotic therapy, invasive angiography, or automatic cardiology referral for every patient. Instead, troponin elevation should prompt selective assessment based on clinical phenotype, baseline risk, symptom profile, ECG findings, biomarker trajectory, and post-discharge stability [21].

Limitations of Evidence

Several limitations warrant cautious interpretation. Only six studies met the focused eligibility criteria, and they represented clinically distinct populations, including type 2 myocardial infarction, critical illness, suspected ACS, and acute or chronic HFrEF [13-18]. Differences in troponin assays, thresholds, sampling schedules, comparators, and outcome definitions limited direct comparison and precluded quantitative meta-analysis. Most findings arose from observational cohorts or secondary biomarker analyses of randomized trials; therefore, the reported associations cannot establish causality or demonstrate that troponin-guided management improves outcomes [14-18]. Residual confounding from illness severity, renal dysfunction, comorbidity burden, and structural heart disease also remains likely [14,17].

At the review level, the restriction to peer-reviewed literature indexed in three databases, absence of grey-literature and trial-registry searches, and lack of prospective protocol registration may have increased the risks of publication and reporting bias. The 2015-2026 date restriction may also have excluded earlier relevant evidence, although it was selected to emphasize contemporary assays and clinical definitions. Consequently, the findings should be regarded as a qualitative synthesis of phenotype-specific prognostic associations rather than support for a uniform management pathway across all forms of non-type 1 myocardial injury [5,20].

Future Directions

Future research should move from risk description toward testing structured responses to troponin elevation outside type 1 myocardial infarction. Prospective studies are needed to determine whether phenotype-based pathways improve outcomes after ACS has been excluded. Such pathways could evaluate whether selected patients benefit from repeat troponin testing, echocardiography, natriuretic peptide measurement, coronary risk assessment, rhythm monitoring, renal evaluation, heart failure optimization, or early post-discharge review. Trials should also clarify which troponin patterns carry actionable risk, including persistent elevation, rising or falling injury patterns, and subthreshold high-sensitivity troponin concentrations [15-18]. The next step is not simply to detect more myocardial injury, but to determine which troponin-positive patients benefit from structured cardiovascular reassessment and targeted follow-up [5,20].

## Conclusions

Cardiac troponin elevation in the absence of type 1 myocardial infarction should not be regarded as a negative ACS workup or an incidental biochemical abnormality. Across type 2 myocardial infarction, critical illness, suspected ACS evaluation, and heart failure populations, troponin elevation consistently identifies patients with greater clinical vulnerability and higher risk of adverse outcomes. Its interpretation should therefore move beyond a binary rule-in or rule-out approach and toward a phenotype-based assessment that considers mechanism, clinical context, biomarker trajectory, and follow-up needs. The central take-home message is that “troponin-positive but not type 1 myocardial infarction” should not mean “low risk” or “not cardiac”; rather, it should prompt structured cardiovascular risk recognition and selective reassessment. Future work should determine which troponin-positive patients benefit most from targeted investigation, therapy optimization, and post-discharge surveillance.
